# Supplementary material for: From body hulls to musculoskeletal models: Personalized inertial parameter estimation
Source: PLoS One. 2026 Jun 24;21(6):e0349886. doi: 10.1371/journal.pone.0349886 (PMC13293452; doi:10.1371/journal.pone.0349886)
Supplement: Attachment — Submitted filename: 2nd_Letter_in_template_v0.pdf [file pone.0349886.s003.pdf]

Xianlian Zhou

Academic Editor,
PLOS ONE

Chair of Autonomous Systems and Mechatronics

Markus Gambietz, M.Sc.

Carl-Thiersch-Straße 2b
91052 Erlangen
+49 9131 85-27 890

markus.gambietz@fau.de
www.mad.tf.fau.de

Erlangen, 17. März 2026

Responses to the reviewer's comments

Dear Dr. Zhou, dear reviewers,

We would like to express our sincere appreciation for the time and effort you have dedicated to re-reviewing our manuscript entitled, "From Body Hulls to Musculoskeletal Models: Personalized Inertial Parameter Estimation".

Below, we provide detailed responses to each of the comments and suggestions made by the reviewers. We have addressed each comment in turn, and the corresponding changes in the manuscript are described. In this document, reviewer comments are in *italic*, and our response is in black. To enhance clarity, we directly included the resulting change in the manuscript where applicable. Changes made based on Reviewer #1's comments are highlighted in blue, Reviewer #2 in green, and magenta based on both reviewers suggested changes. All line numbers refer to the document with marked changes.

We would like to thank you again for letting us submit our revised manuscript and look forward to hearing from you!

Responses to Reviewer #1

I thank the editor and the authors for the opportunity to review this revised manuscript, now renamed to "From Body Hulls to Musculoskeletal Models: Personalized Inertial Parameter Estimation". The revisions have improved the clarity and readability of the manuscript.

1. *The authors state that the "thin-plate spline" radial basis function was replaced with a "cubic" interpolation kernel in the revised version. For complete understanding, it would be helpful to briefly clarify the rationale for selecting the cubic formulation for this application.*
2. *The clarity of the abstract has improved by separating the MRI ground truth validation from the gait experiments.*
3. *The authors have clearly addressed my comments regarding clarification and minor corrections.*
4. *The authors have appropriately addressed my comments and added the requested methodological clarifications and comparative analyses related to internal tissue estimation.*

We thank the reviewer for the positive re-assessment of our study. The "thin-plate spline"-kernel we have used in our initial version takes the form of $r^2 \log(r)$, where r is the distance between points in the interpolated and reference topolopy. We were mistakingly choosing this kernel, which is only suited for even-numbered dimensions. Therefore, we employed a r^3-kernel in the revised version, which is applicable for our 3-dimensional interpolation problem.

[1] M. D. Buhmann, Radial Basis Functions: Theory and Implementations, Cambridge University Press, 2003.

Responses to Reviewer #2

General Comments

The authors have submitted a significantly improved revised manuscript that demonstrates a strong commitment to addressing the concerns raised in the first round of review. The quality of the revision is evident not only in the textual changes but also in the detailed and professional response letter. The decision to change the title from "From Smartphone Images..." to "From Body Hulls..." is particularly commendable, as it accurately reframes the methodological contribution and reduces the risk of misleading readers regarding the role of the acquisition tool. Furthermore, the addition of usability metrics, such as workflow time and expertise requirements, has substantially enhanced the reproducibility and practical value of the study. The response letter is logical, evidence-based, and mostly fully implemented in the revised text. However, there are several presentational aspects regarding data visualization, the explicit statement of limitations, and methodological clarity that

could be further refined to maximize clarity and ensure full compliance with PLOS ONE standards. Therefore, I recommend a Minor Revision to address these remaining points before final acceptance.

We thank the reviewer for the positive evaluation and constructive feedback and appreciate that the revision was well-received. We also thank the reviewer for further suggestions, which have improved the accurate description of our method.

Major Concerns

1. *The authors have appropriately changed the title to "From Body Hulls to Musculoskeletal Models," which mitigates the risk of overemphasizing the smartphone component. However, the Abstract still requires a final check to ensure consistency with this new framing. While the body text has been updated, the Abstract should explicitly mirror the distinction that the smartphone is merely an acquisition tool for the body hull, rather than the core innovation itself. Please ensure the final Abstract strictly aligns with the revised title to avoid any residual confusion for early-career readers.*

We thank the reviewer for this suggestion. We have improved the wording in the abstract so that it states that the body hulls were "acquired via smartphone pictures" instead of "reconstructed from smartphone pictures".

2. *In response to the previous review, the authors added specific runtime data to the Methods section and clarified the expertise required. The distinction regarding the manual MRI labeling time is now explicitly stated, which provides crucial context for comparing SIPP against MRI-derived models. While this response is comprehensive, I recommend ensuring that these usability metrics are highlighted prominently in the Discussion section as well, not just the Methods. This ensures the practical advantage is clearly communicated to the broader readership who may skip detailed methodological descriptions.*

We thank the reviewer for the suggestion. The runtime, required expertise, and manual MRI labeling time are detailed in the Methods and have now been highlighted in the Discussion in line 326-328.

3. *The authors have explicitly distinguished between non-clinical field settings and clinical settings, which was missing in the original draft. This nuance manages user expectations appropriately. However, the manuscript should reinforce the limitation that smartphone-acquired hulls are not intended for clinical diagnostic use without further validation. Please ensure the Conclusion section explicitly reiterates that clinically approved 3D body scanners are preferred for clinical biomechanical analyses, as stated in the response letter, to prevent potential misapplication of the method in medical settings.*

We thank the reviewer for highlighting this point. As suggested, we have explicitly reiterated in the Conclusion section (line 487) that clinical use cases require validated body scanners.

4. *Regarding the quantitative comparison with Lee et al. (2009) and Chang et al. (2018), the authors provided a valid justification for not including direct numerical comparisons due to data availability and metric incompatibility. This is a responsible approach. However, while the explanation in the response letter is sufficient, I recommend ensuring that this limitation is explicitly visible to readers in the final manuscript's Discussion section, rather than just being implied. This ensures transparency for future readers who do not have access to the review history.*

We thank the reviewer for hinting that the limitations of these comparisons were not clearly visible in the manuscript. We have therefore added a sentence that specifies these limitations: "Both Lee et al'.s and Chang et al.'s methods are not directly comparable to our method and dataset, as they either require different data modalities or do not provide an open-source pipeline for BSIP estimation." (ln. 354-356)

5. *The authors clarified the description of trimming the body hull by removing the head and arms in the response letter. Specifically, the definition of "nearest SMPL vertices" based on Euclidean distance is now included. To ensure full reproducibility, please verify that this specific criterion is clearly defined in the final manuscript text itself, not just in the response letter. Any ambiguity here could hinder other researchers from replicating the SIPP pipeline exactly, which is critical for open science standards.*

We thank the reviewer for their comment. The criterion for trimming the body hull (removing the head and arms) based on the nearest SMPL vertices using Euclidean distance was already clearly described in the Methods section (ln. 144-146).

6. *The manuscript states that the study was approved by the ethics committee and that written informed consent was obtained. Please ensure that the Ethics Statement section in the final manuscript explicitly mentions the form of consent and confirms that all methods were carried out in accordance with relevant guidelines and regulations. This is a mandatory requirement for human subject research in PLOS ONE and must be clearly visible in the Methods section.*

We thank the reviewer for pointing out this potential journal guideline violation. The data acquisition section now explicitly states that all procedures were conducted in accordance with the Declaration of Helsinki and that written consent was provided *before* the experiments started (ln. 94-96)

Minor Concerns

1. *There is one area where the response could be improved regarding Figure 4 and the gait cycle phase labels. The authors noted that they tested adding visual markers for the late swing phase but opted to keep the original presentation, arguing that grid lines are sufficient. While I understand the desire for a clean visual presentation, relying solely on grid lines requires extra cognitive load from the reader to map the text description to the visual data. A subtle visual cue, such as a light gray shaded background region between 40-50% on the x-axis, would significantly enhance reader comprehension without cluttering the plot. I suggest the authors reconsider this minor visualization improvement to ensure the key finding is immediately apparent.*

We have revised Figure 4 by adding a subtle shaded region (light gray) to indicate the late swing phase (40–50% of the gait cycle) and indicated the shaded region in the figure caption.

2. *The authors mentioned excluding extreme outliers from the y-axis of Fig. 3 because they are dominated by processing or measurement errors. They added a disclaimer to the figure caption. Please ensure this disclaimer is sufficiently prominent and clearly states the percentage of data excluded (e.g., the top 5%). This prevents readers from questioning whether the plotted data fully reflects the reported reduction in residual forces. Transparency about data exclusion is critical for statistical integrity.*

We thank the reviewer for highlighting that the figure caption needed improvement. The disclaimer in the figure caption now reads: "For visualization purposes, the top 5% of data points (extreme outliers due to processing or measurement errors) are not shown in the y-axis range. Mean values were computed using the full dataset without exclusion."

3. *The manuscript reports participant age, height, and mass with mean. However, additional demographic information would enhance the contextual understanding of the study's generalizability and support the authors' discussion of gender bias and representation. Please consider adding: (1) participant ethnicity or geographic origin, (2) activity level or occupation if relevant to gait patterns, (3) any additional exclusion criteria beyond those mentioned, (4) a table in the Supporting Information with individual participant demographics. This information is particularly relevant given the authors' emphasis on addressing gender bias and the need for diverse representation in biomechanical modeling.*

We thank the reviewer for this important suggestion regarding participant characterization and generalizability. We agree that transparent reporting of demographics is important. Participant age, height, mass, and sex distribution are already reported in the supporting document. Additional variables such as activity level or occupation were not recorded at the time of data collection. Information on ethnicity/geographic origin was not collected, and we note that reporting such data at the individual level in a relatively small cohort

could raise participant re-identification concerns. For this reason, and in accordance with data protection considerations, we do not provide individual-level demographic tables.

4. *The study relies on a relatively small cohort recruited from a single university. While the authors discuss gender bias, the sample size limits the statistical power of these claims. Please ensure the Limitations section explicitly cautions readers against over-generalizing the gender-specific findings (e.g., female models performing better on the benchmark) without validation on larger, more diverse populations. This adds necessary scientific rigor to the claims made in the Discussion regarding bias reduction.*

We agree with the reviewer's comment and have added a disclaimer noting that the gender-specific findings should be interpreted cautiously due to the reduced sample size in these analyses: "however, these findings should be interpreted cautiously, as the sample size is halved in gender-specific analyses." (ln. 438-439)

5. *The manuscript calculates metabolic costs using Kim and Roberts' joint space metabolic model. While this citation is provided, there is brief context regarding why this specific model was chosen over other established metabolic models in the context of personalized inertial parameters. Please add one to two sentences in the Discussion justifying the selection of this metabolic model, particularly regarding its sensitivity to inertial parameter changes and its appropriateness for evaluating BSIP personalization effects.*

We selected the joint-space metabolic model by Kim and Roberts due to its formulation based directly on joint kinematics and kinetics, which avoids additional assumptions associated with muscle-driven models. This joint-space model offers a high repeated measures correlation of >0.9 and is therefore suitable to use in for metabolic cost estimations [1]. We have implemented this information as a footnote (page 9), as the explanation would interfere with readability at this point of the manuscript presentation.

[1] M. Gambietz et al., "Contributing Components of Metabolic Energy Models to Metabolic Cost Estimations in Gait", IEEE TBME, 2024.

Conclusion

The revised manuscript is of high quality and has addressed all major scientific concerns regarding the method's framing, usability, and validity. The authors' responses are logical and well-implemented. The remaining suggestions listed above are aimed at enhancing reader experience, ensuring key findings and limitations are prominently displayed, and meeting specific journal formatting requirements. Specifically, I recommend adding a subtle visual marker in Figure 4, ensuring the limitation regarding external dataset comparisons is explicitly stated in the Discussion text, and verifying the data availability statement

compliance. Once these small adjustments are made, the manuscript will be ready for publication.

We thank the reviewer again for their detailed assessment. We are pleased that the revisions have addressed the major scientific concerns regarding the method's framing, usability, and validity, and that our responses were considered logical and well-implemented.
