# Supplementary material for: From body hulls to musculoskeletal models: Personalized inertial parameter estimation
Source: PLoS One. 2026 Jun 24;21(6):e0349886. doi: 10.1371/journal.pone.0349886 (PMC13293452; doi:10.1371/journal.pone.0349886)
Supplement: Attachment — Submitted filename: Letter_in_template_v1.docx [file pone.0349886.s002.docx]

Xianlian Zhou

*Academic Editor,
PLOS ONE*

**Chair of Autonomous Systems and Mechatronics**

Markus Gambietz, M.Sc.

Carl-Thiersch-Straße 2b

91052 Erlangen

+49 9131 85-27 890

markus.gambietz@fau.de

www.mad.tf.fau.de

Erlangen, 22. Dezember 2025

**Responses to the reviewer’s comments**

Dear Dr. Zhou, dear reviewers,

We would like to express our sincere appreciation for the time and effort you have dedicated to reviewing our manuscript entitled, "From Smartphone Images to Musculoskeletal Models: Personalized Inertial Parameter Estimation".

Below, we provide detailed responses to each of the comments and suggestions made by the reviewers. We have addressed each comment in turn, and the corresponding changes in the manuscript are described. In this document, reviewer comments are in *italic*, and our response is in black. To enhance clarity, we directly included the resulting change in the manuscript where applicable. Changes made based on Reviewer #1’s comments are highlighted in blue, Reviewer #2 in green, and magenta based on both reviewers suggested changes. All line numbers refer to the document with marked changes.

We would like to thank you again for letting us submit our revised manuscript and look forward to hearing from you!

General changes

As a general update, we corrected an oversight in our original pipeline: in line 219, the “thin-plate spline” radial basis function was replaced with a “cubic” interpolation kernel. The thin-plate spline kernel was not suitable for our specific application and resulted in an incorrect implementation of the interpolation. This change only has a marginal effect on the calculated inertial parameters and does not affect the overall conclusions of the study. The results have been updated accordingly.

Responses to Reviewer #1

General Comments

Thank you for your assessment and detailed feedback on our study. We agree with all of your comments regarding the format, language, abstract, introduction, methods, and results, and have implemented the corresponding changes in the manuscript. We also agree with your comments on the discussion and have added further explanations to clarify the changes made in that section.

Format

1. *Please ensure cross references to figures and tables are correctly assigned (Example: Line 232).*
2. *Please ensure proper and consistent labeling of subfigures in accordance with PLOS ONE style. Currently, there is a mix of formats such as “a” and “a).” I believe the correct convention is to use capital letters (A, B, C, ...). Please confirm and apply this consistently.*

Thank you for noticing these format errors. We have fixed the incorrect cross-references and switched to capital letters for figure annotations.

Language

1. *Please stick to either UK or US Engilsh. Your draft uses a mixture of both (colour vs personalized, centre vs center.) which is not recommended.*
2. *Please ensure proper use of Oxford commas (Example: Line 228).*

Thank you for pointing to these issues. We have switched all inconsistent formulations to US english and added missing oxford commas. Changes appear throughout the manuscript.

Abstract

1. *“These personalizated models were validated with an experiment including gait and magnetic resonance imaging measurements.”: Typo – “personalized models”*
2. *The sentence “These personalized models were validated with an experiment including gait and magnetic resonance imaging measurements” is not very clear. ‘gait measurements’ feels vague and could be more precise.*
3. *The final section of the abstract that introduces the two new generic models is not very clear and could be explained more clearly.*

Thank you for these suggestions. We have rearranged the abstract so to clarify the validation procedure and give clearer motivation for the introduction of the new generic models.

Introduction

1. *Line 12: I suggest rewriting the sentence below to accommodate a universal context, as the current definition for residual forces seems to only fit the simple gait-forceplate context. “Then, a motion reconstructed via inverse kinematics (IK) and inverse dynamics (ID) will result in residual forces, which are the difference between the measured ground reaction forces and the external forces estimated by the MSK model.”*

Thank you for the suggestion, we have changed the wording to fit a broader context (“ground reaction forces” 🡪 “external forces”, ln. 14-15).

2. *Line 19: The tool name is widely known as “AddBiomechanics”; for consistency and courtesy to the developers it is best to keep the original format. Applies to all instances of “addBiomechanics” in the paper.*
3. *Line31: Suggesting the usage of comma “e.g.,”*
4. *Line 52: Readers from outside the field might benefit from one brief definition for Skinned Multi-Person Linear (SMPL) model. Or consider providing the full form.*
5. *Line 54: Again readers might not be familiar with SKEL. Even though citation is provided, readers might benefit from a very short explanation inbuilt into the paragraph. e.g., “SKEL, a parametric model that infers skeletal shape and joint positions from body surface.”*
6. *Similarly HIT – Human Implicit Tissues - HIT: Estimating Internal Human Implicit Tissues from the Body Surface. The other option might be to leave out the details (such as tool names) in the introduction section and only detailing them in the Materials and Methods section.*
7. *Line 78-80: “We found that by SIPP and SIPP-generic lead to reduced residuals and that joint torques from SIPP and SIPP-generic would be more similar to MRI-personalized than scaled MSK models.” is not clear. Consider changing it to something like “We found that both SIPP and SIPP-generic reduced residuals, and that their joint torque estimates were more similar to MRI-personalized models than to scaled MSK models.”*

Thaank you for these suggestions, we have applied the suggestions to the respective parts of the introduction (ln. 52-59, 86-89).

Materials and methods

1. *Line 86: Better to spell out “years” in full.*
2. *Lines 88-90: Please update university name in the final version.*
3. *Line 97: Might be good to re-mention the name of the app here again, even though –it was mentioned earlier in the introduction section.*
4. *Line 116: Please add manufacturer details within parentheses for the scanner if possible.*
5. *Line 145: Please follow Plos One guidelines for cross referencing tables.*
6. *Line 150: “We then segment all resulting meshes based planes that we constructed SKEL’s pose and joint centers.” sentence seems to be missing a connecting word.*
7. *Line 199: Title “MRIgait benchmark” can be “MRIgait Benchmark” to follow Title case as is custom with Plos One.*
8. *Line 204: Better to have the abbreviation MRD immediately next to its full form “mean relative deviation”.*
9. *Line 217: BioAmass is typically written as BioAMASS (where AMASS stands for Archive of Motion capture As Surface Shapes)*

Thank you for pointing these out. We have incorporated changes into the manuscript (ln. 98-99; 107; 131; 164; 179; 224; 229; 243)

Results

1. *Line 232: Table cross reference error.*
2. *Line 236: Mixes singular article ‘a’ with plural ‘reductions’*
3. *Table 2 Caption: Typo - “The first two row” ‘s’ missing in ‘rows’*
4. *Line 249: ‘Gait analysis outcomes’ >> ‘Gait Analysis Outcomes’*
5. *Line 257: Please ensure all cross references to Tables and Figures are correctly assigned.*
6. *Line 273: Comma Splice. Better to use a conjunction to join the phrases.*
7. *Figure 6 Caption: Might help to mention that the unfilled circles are outliers*

Thank you for these suggestions. We changed the respective phrases in the manuscript (ln. 256; 261; 276; 300-301; Table 2; Figure 6).

Discussion

1. *More details would be better to briefly summarize the gender based findings in the main paper and discuss how the male and female SIPP-generic models differ in inertial distribution and performance.*

Thank you for this suggestion. We agree that an analysis of sex-specific modeling variations is valuable. However, the sample size per gender in our study is too small to draw definitive conclusions. The most noticeable gender difference appears in the MRIgait benchmark. Female scaled models perform worse than male scaled models, whereas with SIPP, this trend is reversed. This indicated that our method may be particularly effective for female participants. Furthermore, the SIPP(-generic) models show different mass proportions between thigh and torso. We have expanded the discussion regarding these details (ln. 449-455).

2. *Line 298-300: This is the first time the alternative (SMPL only and InsideHumans) are mentioned, please consider adding it to the Methods section and the corresponding residual forces, moments and metabolic reduction in the Results section.*

Thank you for your concern. We have added a section addressing the sensitivity to the internal tissue estimation model in both the Methods (ln. 191-194) and Results (ln. 310-322). To maintain conciseness, we have shortened the corresponding paragraph in the Discussion (ln. 337-349). We chose not to include these additional results in the main figures and tables to not distract from the central narrative of the manuscript. Instead, the detailed results are provided in the supporting material. These additional results are provided exclusively in the supporting material’s tables, since adding the corresponding lines to the plots would overly clutter the figures and render them difficult to interpret.

3. *Line 300: InsideHumans is not a well-known abbreviation or tool name for the layered anatomical model by Komaritzan et al. [15]. Therefore, it would be good to revise the first occurrence to something like: “…where we use the layered anatomical model introduced by Komaritzan et al. [15] (i.e., InsideHumans) instead of HIT.”*
4. *Line 318: It is better to avoid using the term “invasive.” I believe what you mean is that the method is impractical and not easily applicable, as it involves ionizing radiation.*
5. *Line 404: “…by addressing other froms of bias..” typo*

Thank you for bringing up these 3 additional points for the discussion, we have corrected the respective sections in the manuscript (ln.191-194; 361-362; 458).

Responses to Reviewer #2

General Comments

*The study is on the personalization of inertial parameters in musculoskeletal models, as well as providing the field with exploratory content. The novel shape-based inertial parameter personalization method is interesting. Unfortunately, the manuscript does not demonstrate sufficient technical over existing methods. The overall contribution and performance improvement are too limited to warrant publication in its current form.*

Thank you for your critical and detailed assessment of our study. We have addressed your major and minor concerns as listed below.

Regarding your comment that SIPP’s *“performance improvement[s] are too limited,”* we are unsure which specific metrics you are referring to. In the MRIgait benchmark, SIPP reduces mean relative deviations by more than 50% compared to standard scaling across mass, center of mass, moment of inertia, and kinetic energy parameters (addition to ln. 262-263). Similarly, SIPP decreases inverse-dynamics residuals by more than 13% relative to a scaled baseline (unchanged in ln. 277). When combined with the AddBiomechanics workflow, SIPP still provides an additional improvement of more than 10% over AddBiomechanics alone (unchanged ln. 279-282).

We would also like to point out that the achievable lower bound for inverse-dynamics residuals is fundamentally limited by measurement noise and rigid-body modeling assumptions. Therefore, SIPP does not merely reduce residuals by >10% in absolute terms; rather, it moves the solution closer to the practical lower bound imposed by experimental and modeling constraints (addition in ln. 428-431).

Major Concerns

1. *The title “From Smartphone Images to Musculoskeletal Models” and the abstract risk misleading early-career readers or researchers outside the biomechanics field by overemphasizing the role of smartphone images. In fact, the smartphone is only used for body hull reconstruction, while the core of the SIPP method relies on the fusion of multiple specialized models. This title and abstract exaggerate the role of smartphones.*

Thanks for the critical perspective. We changed the title and wording in the abstract to avoid confusion about our contribution.

2. *The study omits two key metrics for SIPP usability, total workflow time and required expertise. It lacks quantification of SIPP’s full-pipeline duration and comparison to MRI-derived MSK models and fails to clarify if key steps need specialized skills, such as proficiency in computational geometry, OpenSim scripting or manual MRI annotation.*

Thank you for this suggestion. We have added a clarification on the runtime of the SIPP pipeline (ln. 186-188), which is about 5 minutes per participant when a GPU is available. As the pipeline is automated, expert knowledge is not needed besides technical experience to set up the python environments. We have also hinted that we are working on a web-app for the SIPP pipeline. The web app would also eliminate the need for technical expertise to set up the Python environments.(ln. 481-484). For manual review and correction of the MRI labels, several hours per participant were required (added in ln. 206). For the labeling of MRI data, expert knowledge is required.

3. *It is unclear on the SIPP method’s target applications and the unique value of smartphone-acquired body hulls. Notably for its proposed clinical biomechanical analysis use, smartphone hulls lack clear rational, MRI offers high-fidelity tissue and skeletal data, and the smartphone step may introduce artifacts needing manual correction without proven accuracy gains over MRI.*

Thank you for this helpful comment. We have added explicit clarifications of the target applications of both SIPP and SIPP-generic in the Introduction (ln. 72-85).

Regarding the role of smartphone-acquired body hulls, we stated that they enable body-shape capture without any specialized equipment (unchanged in ln. 43-45), which makes them suitable for large-scale studies, field settings, and non-clinical environments where bodyscanners or MRI are not feasible.

We would like to clarify that the manuscript does **not** explicitly propose smartphone-based workflows for clinical biomechanical analyses. In fact, we explicitly state that clinically approved 3D body scanners should be used in clinical settings because they provide standardized acquisition procedures and reliable reconstruction of the upper limbs (unchanged in ln. 484–486).

Finally, while MRI remains the gold standard for high-fidelity tissue and skeletal information, but it is costly, time-consuming (both in acquisition and labeling), and typically performed in a supine posture, which requires shape correction when used for musculoskeletal modeling (addition in ln. 206; unchanged in ln. 209-215).

4. *While the study references "personalized reference", the model used lacks sufficient personalized data, with minimal differentiation between female and male individuals, further weakening its personalization.*

Thank you for raising this point. The term “personalized reference” does not appear in our original manuscript, so we believe there may have been some ambiguity in how we described the MRI-based reference models. To clarify: our “reference” musculoskeletal models are fully personalized in their inertial parameters using subject-specific MRI data. These models therefore include individualized segment volumes, mass distributions, and inertial properties for each participant.

5. *The proposed method lacks quantitative comparison with personalized models by Lee et al. 2009 DEXA-based and Chang et al. 2018 3D scan-driven. Only qualitative alignment of conclusions is noted in the Discussion section, with no key data compared, making it hard to assess SIPP’s accuracy advantage.*

Thank you for this suggestion. We agree that a direct quantitative comparison to the methods of Lee et al. (2009) and Chang et al. (2018) would be valuable. However, we were unable to include such comparisons for several reasons:

- **Lack of compatible data:** We do not have DEXA scans for our participants, and neither Lee et al. nor Chang et al. have released their participant-level datasets publicly. As a result, reproducing their personalized inertial parameter estimates is not feasible.
- **Incompatibility of residual-based metrics across datasets:** Quantities such as inverse-dynamics residual forces and moments cannot be meaningfully compared across different cohorts, hardware setups, and marker protocols. (Werling, K., et al. (2023). AddBiomechanics: Automating model scaling, inverse dynamics and standardization. PLOS Comp. Biol.; Fleischmann S., et al. (2025). Exploring Dataset Bias and Scaling Techniques in Multi-Source Gait Biomechanics: An Explainable Machine Learning Approach. ACM TIST)
- **Scope and limitations of prior methods:** Chang et al. (2018) evaluated only the mass distribution for a single participant. Their approach resembles a simplified variant of SIPP without internal tissue estimation, for which we now include detailed data in the appendix for completeness. Furthermore, both Lee et al. (2009) and Chang et al. (2018) do not clearly describe how body-segment boundaries were defined or segmented, and manual segmentation would require expert knowledge and introduce unavoidable variability.

We have corrected an inaccuracy in the corresponding part of the Discussion (ln. 366-369).

Minor Concerns

1. *The manuscript’s hyperlinks contain garbling, which hinders access to target pages and compromises academic standardization.*

Thank you for noticing, we have fixed the incorrect hyperlinks (ln. 499-509).

2. *Fig. 3 notes a "14.9% reduction in residual forces" for SIPP, but the y-axis limits of the figure exclude 5% of outliers without explanation. This raises questions about whether the plotted data fully reflects the reported reduction.*

Thank you for pointing this out. We excluded extreme outliers from the y-axis of Fig. 3 because they are dominated by processing or measurement errors, which are not representative of typical residual forces. We have added a disclaimer to the figure caption to make this explicit.

The reported 14.9% reduction in residual forces is provided in the text and can be verified from the mean values indicated by the grey triangles in Fig. 3. Including the outliers in the plot (shown below) demonstrates that they are a very small fraction of the data and exhibit orders of magnitude higher residuals. In total, more than 2.5 million individual frames are shown across the four plots.

3. *"Contralateral knee flexion moments" in Fig. 4 lack clear labels for gait cycle phases. The text references a "late swing phase" at 40–50% of the gait cycle, which is unmarked on the figure, hindering readers’ ability to link visual data to conclusions.*

Thank you for this suggestion. We tested a version of Fig. 4 highlighting the late swing phase, but we found that the original presentation provided the clearest view of the data. The figure’s grid and x-axis already indicate 40% and 60% of the gait cycle, which corresponds to the late swing phase referenced in the text. We believe this allows readers to identify the relevant region where the joint moments differ, while maintaining a clean and interpretable plot. We have added additional hints in the plot caption to help readers with interpretation of Fig. 4 and Fig. 5.

4. *Key steps in the SIPP pipeline are underdescribed. The process of trimming the body hull by removing the head and arms does not define the exact criteria for identifying “nearest SMPL vertices”，leaving ambiguity about reproducibility.*

Thank you for this comment. We have clarified the description of this step in the SIPP pipeline (ln. 151-155). Specifically, after fitting the SMPL model to the body hull, we prune vertices corresponding to the head and arms. “Nearest” refers to the vertex on the fitted SMPL mesh that has the shortest Euclidean distance to the vertex on the body scan being assigned to a segment. This clarification ensures that the procedure is fully reproducible.

5. *In the Body Hull data acquisition section, the manuscript states that full-body scans of participants were acquired using a smartphone app, with the requirement of "suitable clothing" to ensure scan quality. However, the term "suitable" is not explicitly defined, and no details on clothing standardization are provided.*

Thank you for pointing this out, as the clothing worn during recording is very important for reproducibility. We have included the guideline from our study protocol in context: “(To eliminate clothing artifacts,) participants were asked to wear minimal clothing that is as tight-fitting as possible, but only as far as they felt comfortable.” (ln. 109-111)
