# Supplementary material for: From body hulls to musculoskeletal models: Personalized inertial parameter estimation
Source: PLoS One. 2026 Jun 24;21(6):e0349886. doi: 10.1371/journal.pone.0349886 (PMC13293452; doi:10.1371/journal.pone.0349886)
Supplement: Peer review history

# Peer review: Decision Letter 0

**DOI:** 10.1371/journal.pone.0349886.r001

**Authors:** Xianlian Zhou

7 Nov 2025

PONE-D-25-46723From Smartphone Images to Musculoskeletal Models: Personalized Inertial Parameter EstimationPLOS ONE

Dear Dr. Gambietz,

Thank you for submitting your manuscript to PLOS ONE. After careful consideration, we feel that it has merit but does not fully meet PLOS ONE’s publication criteria as it currently stands. Therefore, we invite you to submit a revised version of the manuscript that addresses the points raised during the review process.

Please submit your revised manuscript by Dec 22 2025 11:59PM. If you will need more time than this to complete your revisions, please reply to this message or contact the journal office at plosone@plos.org. When you're ready to submit your revision, log on to https://www.editorialmanager.com/pone/ and select the 'Submissions Needing Revision' folder to locate your manuscript file.

Please include the following items when submitting your revised manuscript:

- A rebuttal letter that responds to each point raised by the academic editor and reviewer(s). You should upload this letter as a separate file labeled 'Response to Reviewers'.
- A marked-up copy of your manuscript that highlights changes made to the original version. You should upload this as a separate file labeled 'Revised Manuscript with Track Changes'.
- An unmarked version of your revised paper without tracked changes. You should upload this as a separate file labeled 'Manuscript'.

If you would like to make changes to your financial disclosure, please include your updated statement in your cover letter. Guidelines for resubmitting your figure files are available below the reviewer comments at the end of this letter.

If applicable, we recommend that you deposit your laboratory protocols in protocols.io to enhance the reproducibility of your results. Protocols.io assigns your protocol its own identifier (DOI) so that it can be cited independently in the future. For instructions see: https://journals.plos.org/plosone/s/submission-guidelines#loc-laboratory-protocols. Additionally, PLOS ONE offers an option for publishing peer-reviewed Lab Protocol articles, which describe protocols hosted on protocols.io. Read more information on sharing protocols at https://plos.org/protocols?utm_medium=editorial-email&utm_source=authorletters&utm_campaign=protocols.

We look forward to receiving your revised manuscript.

Kind regards,

Xianlian Zhou

Academic Editor

PLOS ONE

Journal Requirements:

When submitting your revision, we need you to address these additional requirements.

1. Please ensure that your manuscript meets PLOS ONE's style requirements, including those for file naming. The PLOS ONE style templates can be found at

https://journals.plos.org/plosone/s/file?id=wjVg/PLOSOne_formatting_sample_main_body.pdf and

https://journals.plos.org/plosone/s/file?id=ba62/PLOSOne_formatting_sample_title_authors_affiliations.pdf.

2. We note that the grant information you provided in the ‘Funding Information’ and ‘Financial Disclosure’ sections do not match.

When you resubmit, please ensure that you provide the correct grant numbers for the awards you received for your study in the ‘Funding Information’ section.

3. We note that you have indicated that there are restrictions to data sharing for this study. For studies involving human research participant data or other sensitive data, we encourage authors to share de-identified or anonymized data. However, when data cannot be publicly shared for ethical reasons, we allow authors to make their data sets available upon request. For information on unacceptable data access restrictions, please see http://journals.plos.org/plosone/s/data-availability#loc-unacceptable-data-access-restrictions.

Before we proceed with your manuscript, please address the following prompts:

a) If there are ethical or legal restrictions on sharing a de-identified data set, please explain them in detail (e.g., data contain potentially identifying or sensitive patient information, data are owned by a third-party organization, etc.) and who has imposed them (e.g., a Research Ethics Committee or Institutional Review Board, etc.). Please also provide contact information for a data access committee, ethics committee, or other institutional body to which data requests may be sent.

b) If there are no restrictions, please upload the minimal anonymized data set necessary to replicate your study findings to a stable, public repository and provide us with the relevant URLs, DOIs, or accession numbers. Please see http://www.bmj.com/content/340/bmj.c181.long for guidelines on how to de-identify and prepare clinical data for publication. For a list of recommended repositories, please see https://journals.plos.org/plosone/s/recommended-repositories. You also have the option of uploading the data as Supporting Information files, but we would recommend depositing data directly to a data repository if possible.

Please update your Data Availability statement in the submission form accordingly.

4. Thank you for stating the following in the Acknowledgments Section of your manuscript:

“This work was funded by the Deutsche Forschungsgemeinschaft (DFG, German Research Foundation) – SFB 1483 – Project-ID 442419336, EmpkinS. This work was also supported with an International Travel Grant by the International Society of Biomechanics (ISB).

We note that you have provided additional information within the Acknowledgements Section that is not currently declared in your Funding Statement. Please note that funding information should not appear in the Acknowledgments section or other areas of your manuscript. We will only publish funding information present in the Funding Statement section of the online submission form.

Please remove any funding-related text from the manuscript and let us know how you would like to update your Funding Statement. Currently, your Funding Statement reads as follows:

“The author(s) received no specific funding for this work.”

Please include your amended statements within your cover letter; we will change the online submission form on your behalf.

5. Please ensure that you refer to Figure 4 & 5 in your text as, if accepted, production will need this reference to link the reader to the figure.

6. We note you have included a table to which you do not refer in the text of your manuscript. Please ensure that you refer to Table 2 in your text; if accepted, production will need this reference to link the reader to the Table

7. If the reviewer comments include a recommendation to cite specific previously published works, please review and evaluate these publications to determine whether they are relevant and should be cited. There is no requirement to cite these works unless the editor has indicated otherwise.

[Note: HTML markup is below. Please do not edit.]

Reviewers' comments:

Reviewer's Responses to Questions

**Comments to the Author**

1. Is the manuscript technically sound, and do the data support the conclusions?

The manuscript must describe a technically sound piece of scientific research with data that supports the conclusions. Experiments must have been conducted rigorously, with appropriate controls, replication, and sample sizes. The conclusions must be drawn appropriately based on the data presented.

Reviewer #1: Yes

Reviewer #2: Partly

**********

2. Has the statistical analysis been performed appropriately and rigorously?

Reviewer #1: Yes

Reviewer #2: Yes

**********

3. Have the authors made all data underlying the findings in their manuscript fully available?

The PLOS Data policy requires authors to make all data underlying the findings described in their manuscript fully available without restriction, with rare exception (please refer to the Data Availability Statement in the manuscript PDF file). The data should be provided as part of the manuscript or its supporting information, or deposited to a public repository. For example, in addition to summary statistics, the data points behind means, medians and variance measures should be available. If there are restrictions on publicly sharing data—e.g. participant privacy or use of data from a third party—those must be specified.

Reviewer #1: Yes

Reviewer #2: No

**********

4. Is the manuscript presented in an intelligible fashion and written in standard English?

PLOS ONE does not copyedit accepted manuscripts, so the language in submitted articles must be clear, correct, and unambiguous. Any typographical or grammatical errors should be corrected at revision, so please note any specific errors here.

Reviewer #1: Yes

Reviewer #2: Yes

**********

5. Review Comments to the Author

Please use the space provided to explain your answers to the questions above. You may also include additional comments for the author, including concerns about dual publication, research ethics, or publication ethics. (Please upload your review as an attachment if it exceeds 20,000 characters)

Reviewer #1: 1. Is the manuscript technically sound, and do the data support the

conclusions?

Yes, the manuscript is technically well implemented, and the data presented support the key

conclusions. The modelling pipeline is well described and validated against MRI-derived inertial

parameters. The observed reductions in residual forces and joint moments align with expectations.

2. Has the statistical analysis been performed appropriately and rigorously?

Yes. Descriptive statistics are provided, and although the subject sample is small, this is typical for

proof-of-concept studies, especially in subject-specific musculoskeletal modeling. The analyses are

appropriate for demonstrating feasibility and for validating the pipeline.

3. Have the authors made all data underlying the findings in their

manuscript fully available?

Yes, the data is made available. The generic models are shared on SimTk. The MRIgait dataset is

available at https://zenodo.org/records/15791380. Please ensure correct URLs are provided. Please

also include the project’s website https://gambimar.github.io/sipp/

4. Is the manuscript presented in an intelligible fashion and written in

standard English?

Yes, for the most part it is well written.

Review Comments to the Author

More General Comments

Format

1. Please ensure cross references to figures and tables are correctly assigned (Example: Line 232).

2. Please ensure proper and consistent labeling of subfigures in accordance with PLOS ONE style.

Currently, there is a mix of formats such as “a” and “a).” I believe the correct convention is to

use capital letters (A, B, C, ...). Please confirm and apply this consistently.

Language

3. Please stick to either UK or US Engilsh. Your draft uses a mixture of both (colour vs

personalized, centre vs center.) which is not recommended.

4. Please ensure proper use of Oxford commas (Example: Line 228).

Abstract

“These personalizated models were validated with an experiment including gait and

magnetic resonance imaging measurements.”

Typo – “personalized models”

The sentence “These personalized models were validated with an experiment including gait and

magnetic resonance imaging measurements” is not very clear. ‘gait measurements’ feels vague

and could be more precise.

The final section of the abstract that introduces the two new generic models is not very clear

and could be explained more clearly.

Introduction

Line12:

I suggest rewriting the sentence below to accommodate a universal context, as the current

definition for residual forces seems to only fit the simple gait-forceplate context.

“Then, a motion reconstructed via inverse kinematics (IK) and inverse dynamics (ID) will result

in residual forces, which are the difference between the measured ground reaction forces and

the external forces estimated by the MSK model.”

Line19:

The tool name is widely known as “AddBiomechanics”; for consistency and courtesy to the

developers it is best to keep the original format. Applies to all instances of “addBiomechanics”

in the paper.

Line31:

Suggesting the usage of comma “e.g.,”

Line 52:

Readers from outside the field might benefit from one brief definition for Skinned Multi-Person

Linear (SMPL) model. Or consider providing the full form.

Line 54:

Again readers might not be familiar with SKEL. Even though citation is provided, readers

might benefit from a very short explanation inbuilt into the paragraph. e.g., “SKEL, a

parametric model that infers skeletal shape and joint positions from body surface.”

Similarly HIT – Human Implicit Tissues

HIT: Estimating Internal Human Implicit Tissues from the Body Surface.

The other option might be to leave out the details (such as tool names) in the introduction

section and only detailing them in the Materials and Methods section.

Line 78-80:

“We found that by SIPP and SIPP-generic lead to reduced residuals and that joint torques from

SIPP and SIPP-generic would be more similar to MRI-personalized than scaled MSK models.”

is not clear.

Consider changing it to something like “We found that both SIPP and SIPP-generic reduced

residuals, and that their joint torque estimates were more similar to MRI-personalized models

than to scaled MSK models.”

Materials and methods

Line 86:

Better to spell out “years” in full.

Lines 88-90:

Please update university name in the final version.

Line 97:

Might be good to re-mention the name of the app here again, even though it was mentioned

earlier in the introduction section.

Line 116:

Please add manufacturer details within parentheses for the scanner if possible.

Line 145:

Please follow Plos One guidelines for cross referencing tables.

Line 150:

“We then segment all resulting meshes based planes that we constructed SKEL’s pose and joint

centers.” sentence seems to be missing a connecting word.

Line 199:

Title “MRIgait benchmark” can be “MRIgait Benchmark” to follow Title case as is custom with

Plos One.

Line 204:

Better to have the abbreviation MRD immediately next to its full form “mean relative

deviation”.

Line 217:

BioAmass is typically written as BioAMASS (where AMASS stands for Archive of Motion

capture As Surface Shapes)

Results

Line 232:

Table cross reference error.

Line 236:

Mixes singular article ‘a’ with plural ‘reductions’

Table 2 Caption:

Typo - “The first two row” ‘s’ missing in ‘rows’

Line 249:

‘Gait analysis outcomes’ >> ‘Gait Analysis Outcomes’

Line 257:

Please ensure all cross references to Tables and Figures are correctly assigned.

Line 273:

Comma Splice. Better to use a conjunction to join the phrases.

Figure 6 Caption:

Might help to mention that the unfilled circles are outliers

Discussion

More details would be better to briefly summarize the gender based findings in the main paper

and discuss how the male and female SIPP-generic models differ in inertial distribution and

performance.

Line 298-300:

This is the first time the alternative (SMPL only and InsideHumans) are mentioned, please

consider adding it to the Methods section and the corresponding residual forces, moments and

metabolic reduction in the Results section.

Line 300:

InsideHumans is not a well-known abbreviation or tool name for the layered anatomical model

by Komaritzan et al. [15]. Therefore, it would be good to revise the first occurrence to

something like:

“…where we use the layered anatomical model introduced by Komaritzan et al. [15] (i.e.,

InsideHumans) instead of HIT.”

Line 318:

It is better to avoid using the term “invasive.” I believe what you mean is that the method is

impractical and not easily applicable, as it involves ionizing radiation.

Line 404:

“…by addressing other froms of bias..” typo

Reviewer #2: The study is on the personalization of inertial parameters in musculoskeletal models, as well as providing the field with exploratory content. The novel shape-based inertial parameter personalization method is interesting. Unfortunately, the manuscript does not demonstrate sufficient technical over existing methods. The overall contribution and performance improvement are too limited to warrant publication in its current form.

Major Concerns:

The title“From Smartphone Images to Musculoskeletal Models”and the abstract risk misleading early-career readers or researchers outside the biomechanics field by overemphasizing the role of smartphone images. In fact，the smartphone is only used for body hull reconstruction, while the core of the SIPP method relies on the fusion of multiple specialized models. This title and abstract exaggerate the role of smartphones.

The study omits two key metrics for SIPP usability, total workflow time and required expertise. It lacks quantification of SIPP’s full-pipeline duration and comparison to MRI-derived MSK models and fails to clarify if key steps need specialized skills, such as proficiency in computational geometry, OpenSim scripting or manual MRI annotation.

It is unclear on the SIPP method’s target applications and the unique value of smartphone-acquired body hulls. Notably for its proposed clinical biomechanical analysis use, smartphone hulls lack clear rational, MRI offers high-fidelity tissue and skeletal data, and the smartphone step may introduce artifacts needing manual correction without proven accuracy gains over MRI.

While the study references "personalized reference", the model used lacks sufficient personalized data, with minimal differentiation between female and male individuals, further weakening its personalization.

The proposed method lacks quantitative comparison with personalized models by Lee et al. 2009 DEXA-based and Chang et al. 2018 3D scan-driven. Only qualitative alignment of conclusions is noted in the Discussion section, with no key data compared, making it hard to assess SIPP’s accuracy advantage .

Minor Concerns:

The manuscript’s hyperlinks contain garbling, which hinders access to target pages and compromises academic standardization.

Fig. 3 notes a "14.9% reduction in residual forces" for SIPP, but the y-axis limits of the figure exclude 5% of outliers without explanation. This raises questions about whether the plotted data fully reflects the reported reduction.

"Contralateral knee flexion moments" in Fig. 4 lack clear labels for gait cycle phases. The text references a "late swing phase" at 40–50% of the gait cycle, which is unmarked on the figure, hindering readers’ ability to link visual data to conclusions.

Key steps in the SIPP pipeline are underdescribed. The process of trimming the body hull by removing the head and arms does not define the exact criteria for identifying “nearest SMPL vertices”，leaving ambiguity about reproducibility.

In the Body Hull data acquisition section, the manuscript states that full-body scans of participants were acquired using a smartphone app, with the requirement of "suitable clothing" to ensure scan quality. However, the term "suitable" is not explicitly defined, and no details on clothing standardization are provided.

**********

6. PLOS authors have the option to publish the peer review history of their article (what does this mean?). If published, this will include your full peer review and any attached files.

If you choose “no”, your identity will remain anonymous but your review may still be made public.

**Do you want your identity to be public for this peer review?** For information about this choice, including consent withdrawal, please see our Privacy Policy.

Reviewer #1: No

Reviewer #2: No

**********

[NOTE: If reviewer comments were submitted as an attachment file, they will be attached to this email and accessible via the submission site. Please log into your account, locate the manuscript record, and check for the action link "View Attachments". If this link does not appear, there are no attachment files.]

To ensure your figures meet our technical requirements, please review our figure guidelines: https://journals.plos.org/plosone/s/figures

You may also use PLOS’s free figure tool, NAAS, to help you prepare publication quality figures: https://journals.plos.org/plosone/s/figures#loc-tools-for-figure-preparation.

NAAS will assess whether your figures meet our technical requirements by comparing each figure against our figure specifications.

# Author comment: Author response to Decision Letter 1

**DOI:** 10.1371/journal.pone.0349886.r002

7 Jan 2026

Please find the comments in the attached rebuttal letter at the end of the compiled pdf.

Attachment Submitted filename: Letter_in_template_v1.docx

# Peer review: Decision Letter 1

**DOI:** 10.1371/journal.pone.0349886.r003

**Authors:** Xianlian Zhou

11 Mar 2026

PONE-D-25-46723R1From Body Hulls to Musculoskeletal Models: Personalized Inertial Parameter EstimationPLOS One

Dear Dr. Gambietz,

Thank you for submitting your manuscript to PLOS ONE. After careful consideration, we feel that it has merit but does not fully meet PLOS ONE’s publication criteria as it currently stands. Therefore, we invite you to submit a revised version of the manuscript that addresses the points raised during the review process.

One reviewer recommended the acceptance of your paper with just a minor comment.The second reviewer recommended minor revision with a few points to improve the paper.

Please submit your revised manuscript by Apr 25 2026 11:59PM. If you will need more time than this to complete your revisions, please reply to this message or contact the journal office at plosone@plos.org. When you're ready to submit your revision, log on to https://www.editorialmanager.com/pone/ and select the 'Submissions Needing Revision' folder to locate your manuscript file.

Please include the following items when submitting your revised manuscript:

- A letter that responds to each point raised by the academic editor and reviewer(s). You should upload this letter as a separate file labeled 'Response to Reviewers'.
- A marked-up copy of your manuscript that highlights changes made to the original version. You should upload this as a separate file labeled 'Revised Manuscript with Track Changes'.
- An unmarked version of your revised paper without tracked changes. You should upload this as a separate file labeled 'Manuscript'.

If you would like to make changes to your financial disclosure, please include your updated statement in your cover letter. Guidelines for resubmitting your figure files are available below the reviewer comments at the end of this letter.

If applicable, we recommend that you deposit your laboratory protocols in protocols.io to enhance the reproducibility of your results. Protocols.io assigns your protocol its own identifier (DOI) so that it can be cited independently in the future. For instructions see: https://journals.plos.org/plosone/s/submission-guidelines#loc-laboratory-protocols. Additionally, PLOS ONE offers an option for publishing peer-reviewed Lab Protocol articles, which describe protocols hosted on protocols.io. Read more information on sharing protocols at https://plos.org/protocols?utm_medium=editorial-email&utm_source=authorletters&utm_campaign=protocols.

We look forward to receiving your revised manuscript.

Kind regards,

Xianlian Zhou

Academic Editor

PLOS One

Journal Requirements:

If the reviewer comments include a recommendation to cite specific previously published works, please review and evaluate these publications to determine whether they are relevant and should be cited. There is no requirement to cite these works unless the editor has indicated otherwise.

Please review your reference list to ensure that it is complete and correct. If you have cited papers that have been retracted, please include the rationale for doing so in the manuscript text, or remove these references and replace them with relevant current references. Any changes to the reference list should be mentioned in the rebuttal letter that accompanies your revised manuscript. If you need to cite a retracted article, indicate the article’s retracted status in the References list and also include a citation and full reference for the retraction notice.

[Note: HTML markup is below. Please do not edit.]

Reviewers' comments:

Reviewer's Responses to Questions

**Comments to the Author**

1. If the authors have adequately addressed your comments raised in a previous round of review and you feel that this manuscript is now acceptable for publication, you may indicate that here to bypass the “Comments to the Author” section, enter your conflict of interest statement in the “Confidential to Editor” section, and submit your "Accept" recommendation.

Reviewer #1: All comments have been addressed

Reviewer #2: All comments have been addressed

**********

2. Is the manuscript technically sound, and do the data support the conclusions?

The manuscript must describe a technically sound piece of scientific research with data that supports the conclusions. Experiments must have been conducted rigorously, with appropriate controls, replication, and sample sizes. The conclusions must be drawn appropriately based on the data presented.

Reviewer #1: Yes

Reviewer #2: Partly

**********

3. Has the statistical analysis been performed appropriately and rigorously?

Reviewer #1: N/A

Reviewer #2: Yes

**********

4. Have the authors made all data underlying the findings in their manuscript fully available?

The PLOS Data policy requires authors to make all data underlying the findings described in their manuscript fully available without restriction, with rare exception (please refer to the Data Availability Statement in the manuscript PDF file). The data should be provided as part of the manuscript or its supporting information, or deposited to a public repository. For example, in addition to summary statistics, the data points behind means, medians and variance measures should be available. If there are restrictions on publicly sharing data—e.g. participant privacy or use of data from a third party—those must be specified.

Reviewer #1: Yes

Reviewer #2: Yes

**********

5. Is the manuscript presented in an intelligible fashion and written in standard English?

PLOS ONE does not copyedit accepted manuscripts, so the language in submitted articles must be clear, correct, and unambiguous. Any typographical or grammatical errors should be corrected at revision, so please note any specific errors here.

Reviewer #1: Yes

Reviewer #2: Yes

**********

6. Review Comments to the Author

Please use the space provided to explain your answers to the questions above. You may also include additional comments for the author, including concerns about dual publication, research ethics, or publication ethics. (Please upload your review as an attachment if it exceeds 20,000 characters)

Reviewer #1: General Assessment on the Revised Manuscript

I thank the editor and the authors for the opportunity to review this revised manuscript, now renamed to “From Body Hulls to Musculoskeletal Models: Personalized Inertial Parameter Estimation”. The revisions have improved the clarity and readability of the manuscript.

1. The authors state that the “thin-plate spline” radial basis function was replaced with a “cubic” interpolation kernel in the revised version. For complete understanding, it would be helpful to briefly clarify the rationale for selecting the cubic formulation for this application.

2. The clarity of the abstract has improved by separating the MRI ground truth validation from the gait experiments.

3. The authors have clearly addressed my comments regarding clarification and minor corrections.

4. The authors have appropriately addressed my comments and added the requested methodological clarifications and comparative analyses related to internal tissue estimation.

Reviewer #2: PLOS One：

General Comments

The authors have submitted a significantly improved revised manuscript that demonstrates a strong commitment to addressing the concerns raised in the first round of review. The quality of the revision is evident not only in the textual changes but also in the detailed and professional response letter. The decision to change the title from "From Smartphone Images..." to "From Body Hulls..." is particularly commendable, as it accurately reframes the methodological contribution and reduces the risk of misleading readers regarding the role of the acquisition tool. Furthermore, the addition of usability metrics, such as workflow time and expertise requirements, has substantially enhanced the reproducibility and practical value of the study. The response letter is logical, evidence-based, and mostly fully implemented in the revised text. However, there are several presentational aspects regarding data visualization, the explicit statement of limitations, and methodological clarity that could be further refined to maximize clarity and ensure full compliance with PLOS ONE standards. Therefore, I recommend a Minor Revision to address these remaining points before final acceptance.

Major Concerns

1. The authors have appropriately changed the title to "From Body Hulls to Musculoskeletal Models," which mitigates the risk of overemphasizing the smartphone component. However, the Abstract still requires a final check to ensure consistency with this new framing. While the body text has been updated, the Abstract should explicitly mirror the distinction that the smartphone is merely an acquisition tool for the body hull, rather than the core innovation itself. Please ensure the final Abstract strictly aligns with the revised title to avoid any residual confusion for early-career readers.

2. In response to the previous review, the authors added specific runtime data to the Methods section and clarified the expertise required. The distinction regarding the manual MRI labeling time is now explicitly stated, which provides crucial context for comparing SIPP against MRI-derived models. While this response is comprehensive, I recommend ensuring that these usability metrics are highlighted prominently in the Discussion section as well, not just the Methods. This ensures the practical advantage is clearly communicated to the broader readership who may skip detailed methodological descriptions.

3. The authors have explicitly distinguished between non-clinical field settings and clinical settings, which was missing in the original draft. This nuance manages user expectations appropriately. However, the manuscript should reinforce the limitation that smartphone-acquired hulls are not intended for clinical diagnostic use without further validation. Please ensure the Conclusion section explicitly reiterates that clinically approved 3D body scanners are preferred for clinical biomechanical analyses, as stated in the response letter, to prevent potential misapplication of the method in medical settings.

4. Regarding the quantitative comparison with Lee et al. (2009) and Chang et al. (2018), the authors provided a valid justification for not including direct numerical comparisons due to data availability and metric incompatibility. This is a responsible approach. However, while the explanation in the response letter is sufficient, I recommend ensuring that this limitation is explicitly visible to readers in the final manuscript's Discussion section, rather than just being implied. This ensures transparency for future readers who do not have access to the review history.

5. The authors clarified the description of trimming the body hull by removing the head and arms in the response letter. Specifically, the definition of "nearest SMPL vertices" based on Euclidean distance is now included. To ensure full reproducibility, please verify that this specific criterion is clearly defined in the final manuscript text itself, not just in the response letter. Any ambiguity here could hinder other researchers from replicating the SIPP pipeline exactly, which is critical for open science standards.

6.The manuscript states that the study was approved by the ethics committee and that written informed consent was obtained. Please ensure that the Ethics Statement section in the final manuscript explicitly mentions the form of consent and confirms that all methods were carried out in accordance with relevant guidelines and regulations. This is a mandatory requirement for human subject research in PLOS ONE and must be clearly visible in the Methods section.

Minor Concerns

1. There is one area where the response could be improved regarding Figure 4 and the gait cycle phase labels. The authors noted that they tested adding visual markers for the late swing phase but opted to keep the original presentation, arguing that grid lines are sufficient. While I understand the desire for a clean visual presentation, relying solely on grid lines requires extra cognitive load from the reader to map the text description to the visual data. A subtle visual cue, such as a light gray shaded background region between 40-50% on the x-axis, would significantly enhance reader comprehension without cluttering the plot. I suggest the authors reconsider this minor visualization improvement to ensure the key finding is immediately apparent.

2. The authors mentioned excluding extreme outliers from the y-axis of Fig. 3 because they are dominated by processing or measurement errors. They added a disclaimer to the figure caption. Please ensure this disclaimer is sufficiently prominent and clearly states the percentage of data excluded (e.g., the top 5%). This prevents readers from questioning whether the plotted data fully reflects the reported reduction in residual forces. Transparency about data exclusion is critical for statistical integrity.

3. The manuscript reports participant age, height, and mass with mean. However, additional demographic information would enhance the contextual understanding of the study's generalizability and support the authors' discussion of gender bias and representation. Please consider adding: (1) participant ethnicity or geographic origin, (2) activity level or occupation if relevant to gait patterns, (3) any additional exclusion criteria beyond those mentioned, (4) a table in the Supporting Information with individual participant demographics. This information is particularly relevant given the authors' emphasis on addressing gender bias and the need for diverse representation in biomechanical modeling.

4. The study relies on a relatively small cohort recruited from a single university. While the authors discuss gender bias, the sample size limits the statistical power of these claims. Please ensure the Limitations section explicitly cautions readers against over-generalizing the gender-specific findings (e.g., female models performing better on the benchmark) without validation on larger, more diverse populations. This adds necessary scientific rigor to the claims made in the Discussion regarding bias reduction.

5. The manuscript calculates metabolic costs using Kim and Roberts' joint space metabolic model. While this citation is provided, there is brief context regarding why this specific model was chosen over other established metabolic models in the context of personalized inertial parameters. Please add one to two sentences in the Discussion justifying the selection of this metabolic model, particularly regarding its sensitivity to inertial parameter changes and its appropriateness for evaluating BSIP personalization effects.

The revised manuscript is of high quality and has addressed all major scientific concerns regarding the method's framing, usability, and validity. The authors' responses are logical and well-implemented. The remaining suggestions listed above are aimed at enhancing reader experience, ensuring key findings and limitations are prominently displayed, and meeting specific journal formatting requirements. Specifically, I recommend adding a subtle visual marker in Figure 4, ensuring the limitation regarding external dataset comparisons is explicitly stated in the Discussion text, and verifying the data availability statement compliance. Once these small adjustments are made, the manuscript will be ready for publication.

**********

7. PLOS authors have the option to publish the peer review history of their article (what does this mean?). If published, this will include your full peer review and any attached files.

If you choose “no”, your identity will remain anonymous but your review may still be made public.

**Do you want your identity to be public for this peer review?** For information about this choice, including consent withdrawal, please see our Privacy Policy.

Reviewer #1: No

Reviewer #2: No

**********

[NOTE: If reviewer comments were submitted as an attachment file, they will be attached to this email and accessible via the submission site. Please log into your account, locate the manuscript record, and check for the action link "View Attachments". If this link does not appear, there are no attachment files.]

To ensure your figures meet our technical requirements, please review our figure guidelines: https://journals.plos.org/plosone/s/figures

You may also use PLOS’s free figure tool, NAAS, to help you prepare publication quality figures: https://journals.plos.org/plosone/s/figures#loc-tools-for-figure-preparation.

NAAS will assess whether your figures meet our technical requirements by comparing each figure against our figure specifications.

# Author comment: Author response to Decision Letter 2

**DOI:** 10.1371/journal.pone.0349886.r004

19 Mar 2026

Dear Reviewers,

thanks you for your time and effort. Please find our responses in the attached rebuttal letter.

Kind regards,

Markus Gambietz

Attachment Submitted filename: 2nd_Letter_in_template_v0.pdf

# Peer review: Decision Letter 2

**DOI:** 10.1371/journal.pone.0349886.r005

**Authors:** Xianlian Zhou

6 May 2026

From Body Hulls to Musculoskeletal Models: Personalized Inertial Parameter Estimation

PONE-D-25-46723R2

Dear Dr. Gambietz,

We’re pleased to inform you that your manuscript has been judged scientifically suitable for publication and will be formally accepted for publication once it meets all outstanding technical requirements.

Within one week, you’ll receive an e-mail detailing the required amendments. When these have been addressed, you’ll receive a formal acceptance letter and your manuscript will be scheduled for publication.

An invoice will be generated when your article is formally accepted. Please note, if your institution has a publishing partnership with PLOS and your article meets the relevant criteria, all or part of your publication costs will be covered. Please make sure your user information is up-to-date by logging into Editorial Manager at Editorial Manager® and clicking the ‘Update My Information' link at the top of the page. For questions related to billing, please contact billing support.

If your institution or institutions have a press office, please notify them about your upcoming paper to help maximize its impact. If they’ll be preparing press materials, please inform our press team as soon as possible -- no later than 48 hours after receiving the formal acceptance. Your manuscript will remain under strict press embargo until 2 pm Eastern Time on the date of publication. For more information, please contact onepress@plos.org.

Kind regards,

Xianlian Zhou

Academic Editor

PLOS One

Additional Editor Comments (optional):

Reviewers' comments:

Reviewer's Responses to Questions

**Comments to the Author**

1. If the authors have adequately addressed your comments raised in a previous round of review and you feel that this manuscript is now acceptable for publication, you may indicate that here to bypass the “Comments to the Author” section, enter your conflict of interest statement in the “Confidential to Editor” section, and submit your "Accept" recommendation.

Reviewer #1: All comments have been addressed

Reviewer #2: All comments have been addressed

**********

2. Is the manuscript technically sound, and do the data support the conclusions?

The manuscript must describe a technically sound piece of scientific research with data that supports the conclusions. Experiments must have been conducted rigorously, with appropriate controls, replication, and sample sizes. The conclusions must be drawn appropriately based on the data presented.

Reviewer #1: Yes

Reviewer #2: Yes

**********

3. Has the statistical analysis been performed appropriately and rigorously?

Reviewer #1: Yes

Reviewer #2: Yes

**********

4. Have the authors made all data underlying the findings in their manuscript fully available?

The PLOS Data policy requires authors to make all data underlying the findings described in their manuscript fully available without restriction, with rare exception (please refer to the Data Availability Statement in the manuscript PDF file). The data should be provided as part of the manuscript or its supporting information, or deposited to a public repository. For example, in addition to summary statistics, the data points behind means, medians and variance measures should be available. If there are restrictions on publicly sharing data—e.g. participant privacy or use of data from a third party—those must be specified.

Reviewer #1: Yes

Reviewer #2: Yes

**********

5. Is the manuscript presented in an intelligible fashion and written in standard English?

PLOS ONE does not copyedit accepted manuscripts, so the language in submitted articles must be clear, correct, and unambiguous. Any typographical or grammatical errors should be corrected at revision, so please note any specific errors here.

Reviewer #1: Yes

Reviewer #2: Yes

**********

6. Review Comments to the Author

Please use the space provided to explain your answers to the questions above. You may also include additional comments for the author, including concerns about dual publication, research ethics, or publication ethics. (Please upload your review as an attachment if it exceeds 20,000 characters)

Reviewer #1: (No Response)

Reviewer #2: (No Response)

**********

7. PLOS authors have the option to publish the peer review history of their article (what does this mean?). If published, this will include your full peer review and any attached files.

If you choose “no”, your identity will remain anonymous but your review may still be made public.

**Do you want your identity to be public for this peer review?** For information about this choice, including consent withdrawal, please see our Privacy Policy.

Reviewer #1: No

Reviewer #2: No

**********

# Editor report: Acceptance letter

**DOI:** 10.1371/journal.pone.0349886.r006

**Authors:** Xianlian Zhou

PONE-D-25-46723R2

PLOS One

Dear Dr. Gambietz,

I'm pleased to inform you that your manuscript has been deemed suitable for publication in PLOS One. Congratulations! Your manuscript is now being handed over to our production team.

At this stage, our production department will prepare your paper for publication. This includes ensuring the following:

* All references, tables, and figures are properly cited

* All relevant supporting information is included in the manuscript submission,

* There are no issues that prevent the paper from being properly typeset

You will receive further instructions from the production team, including instructions on how to review your proof when it is ready. Please keep in mind that we are working through a large volume of accepted articles, so please give us a few days to review your paper and let you know the next and final steps.

Lastly, if your institution or institutions have a press office, please let them know about your upcoming paper now to help maximize its impact. If they'll be preparing press materials, please inform our press team within the next 48 hours. Your manuscript will remain under strict press embargo until 2 pm Eastern Time on the date of publication. For more information, please contact onepress@plos.org.

You will receive an invoice from PLOS for your publication fee after your manuscript has reached the completed accept phase. If you receive an email requesting payment before acceptance or for any other service, this may be a phishing scheme. Learn how to identify phishing emails and protect your accounts at https://explore.plos.org/phishing.

If we can help with anything else, please email us at customercare@plos.org.

Thank you for submitting your work to PLOS ONE and supporting open access.

Kind regards,

PLOS ONE Editorial Office Staff

on behalf of

Dr. Xianlian Zhou

Academic Editor

PLOS One
